# Peripheral Nerve Diffusion Tensor Imaging: Assessment of Axon and Myelin Sheath Integrity

**DOI:** 10.1371/journal.pone.0130833

**Published:** 2015-06-26

**Authors:** A. Heckel, M. Weiler, A. Xia, M. Ruetters, M. Pham, M. Bendszus, S. Heiland, P. Baeumer

**Affiliations:** 1 Department of Neuroradiology, Heidelberg University Hospital, Heidelberg, Germany; 2 Department of Diagnostic Radiology, Freiburg University Hospital, Freiburg, Germany; 3 Department of Neurology, Heidelberg University Hospital, Heidelberg, Germany; 4 Clinical Cooperation Unit Neurooncology, German Cancer Research Center (DKFZ), Heidelberg, Germany; 5 Section of Experimental Neuroradiology, Department of Neuroradiology, Heidelberg University Hospital, Heidelberg, Germany; The First affiliated Hospital of Xi’an Jiaotong University, CHINA

## Abstract

**Purpose:**

To investigate the potential of diffusion tensor imaging (DTI) parameters as *in-vivo* biomarkers of axon and myelin sheath integrity of the median nerve in the carpal tunnel as validated by correlation with electrophysiology.

**Methods:**

MRI examinations at 3T including DTI were conducted on wrists in 30 healthy subjects. After manual segmentation of the median nerve quantitative analysis of fractional anisotropy (FA) as well as axial, radial and mean diffusivity (AD, RD, and MD) was carried out. Pairwise Pearson correlations with electrophysiological parameters comprising sensory nerve action potential (SNAP) and compound muscle action potential (CMAP) as markers of axon integrity, and distal motor latency (dml) and sensory nerve conduction velocity (sNCV) as markers of myelin sheath integrity were computed. The significance criterion was set at P=0.05, Bonferroni corrected for multiple comparisons.

**Results:**

DTI parameters showed a distinct proximal-to-distal profile with FA, MD, and RD extrema coinciding in the center of the carpal tunnel. AD correlated with CMAP (r=0.50, p=0.04, Bonf. corr.) but not with markers of myelin sheath integrity. RD correlated with sNCV (r=-0.53, p=0.02, Bonf. corr.) but not with markers of axon integrity. FA correlated with dml (r=-0.63, p=0.002, Bonf. corr.) and sNCV (r=0.68, p=0.001, Bonf. corr.) but not with markers of axon integrity.

**Conclusion:**

AD reflects axon integrity, while RD (and FA) reflect myelin sheath integrity as validated by correlation with electrophysiology. DTI parameters consistently indicate a slight decrease of structural integrity in the carpal tunnel as a physiological site of median nerve entrapment. DTI is particularly sensitive, since these findings are observed in healthy participants. Our results encourage future studies to evaluate the potential of DTI in differentiating axon from myelin sheath injury in patients with manifest peripheral neuropathies.

## Introduction

Magnetic Resonance Neurography (MRN) in current practice relies on the assessment of intraneural T2-w contrast and nerve caliber for the assessment of peripheral neuropathy [1]. Nerve T2 signal has previously been shown to have a high sensitivity and diagnostic accuracy [2,3]. As a promising new technique in MRN, Diffusion Tensor Imaging (DTI) is increasingly being utilized to investigate peripheral nerve integrity [4–7].

DTI is based on the diffusion of free water protons along multiple directions in space to characterize tissue microstructure [8]. Fractional anisotropy (FA) is a common read-out parameter indicating the directional preference of diffusion with physiologically high values in intact peripheral nerves (reviewed in [9,10]). Studies on peripheral nerve DTI have demonstrated its general feasibility [10–14] in accurately visualizing nerves by FA. A number of studies applied DTI in carpal tunnel syndrome [15–20]. Recently, we have shown that FA is sufficiently sensitive to detect subclinical lesions of the ulnar nerve at the elbow [21].

In DTI, FA and MD (mean diffusivity) are commonly used summary parameters that represent a simplified description of water diffusion. A more complete account is given by additional evaluation of diffusivity parallel and perpendicular to fiber orientation, also referred to as axial and radial diffusivity (AD and RD), respectively. There is solid evidence in animal studies of the CNS that AD and RD correspond to the axon and myelin sheath integrity of white matter, respectively [22–29]. Concordantly, human studies on multiple sclerosis suggest that axonal damage and demyelination are reflected by changes in AD and RD, respectively (reviewed in [30]). However, the role of AD and RD in the peripheral nervous system in humans with respect to nerval compartments has not been investigated.

Lesion detection in the peripheral nervous system on a microstructural level is important, since it could offer insights into pathophysiological processes enabling early diagnosis and monitoring of peripheral nerve injury [24]. Electrodiagnostics remain the gold standard in non-invasive differentiation of axon and myelin sheath integrity but usually integrate larger nerve segments and are difficult to conduct in anatomical locations that are not easily accessible. Generally, the parameters compound muscle action potential (CMAP) and sensory nerve action potential (SNAP) are considered to probe axon integrity, whereas nerve conduction velocities (NCV) and distal motor latency (dml) probe myelin sheath integrity. While this distinction should not be considered absolute, it is generally in line with histological assessments [31]. How DTI relates to electrophysiological parameters of axon and myelin sheath integrity has not been investigated yet.

In the present study, we investigated the potential of DTI parameters (FA, MD, AD, and RD) as biomarkers of axon and myelin sheath integrity as validated by correlation with electroneurographic parameters. We used the median nerve in the carpal tunnel of healthy volunteers, since the carpal tunnel is well-characterized in nerve conduction studies and represents a typical site of focal nerve pathology. We hypothesized that AD correlates with electrophysiological parameters indicating axon integrity, while RD correlates with electrophysiological parameters indicating myelin sheath integrity.

## Materials and Methods

### Participant data

The study was approved by the institutional ethics board (University of Heidelberg ethics committee; S-057/2009) and written informed consent was obtained from all participants. Participants were examined at the Department of Neuroradiology at Heidelberg University Hospital, Germany. Thirty healthy participants (17 female, 13 male, age range 23–68 years, mean age 39.3 ±15.5 years) without a history of neuropathy were included in the study. Upper extremity pain, hypesthesia or paresthesia, diabetes mellitus or other risk factors of polyneuropathy and signs of median nerve entrapment were excluded by interview (PB, MR).

### MRN Imaging

Examinations were performed on a 3 Tesla unit (Magnetom TIM Trio, Siemens AG, Erlangen, Germany). Standard T2-w images and diffusion-weighted echo-planar-imaging sequences were acquired and covered the distal forearm and wrist (left: 3; right: 27). Subjects were examined in prone position in a dedicated 8-channel wrist-coil. For DTI, we customized a spin-echo echo-planar-imaging sequence from the advanced Diffusion work-in-progress package (ASP 511 E) developed by Siemens. The sequence was optimized for peripheral nerve imaging by increasing spatial resolution keeping acquisition time limited in order to minimize motion artifacts, and choosing the best suited fat suppression technique (spectrally adiabatic inversion recovery—SPAIR) as described previously [21]. Sequence parameters were: TR 3800 ms, TE 99 ms, b-value 0 and 1200 s/mm^2^ (encoded in monopolar 19 directions), readout bandwidth 1395 Hz/px, 18 slices, interslice gap 1.2 mm, FoV 150 x 150 mm^2^, acquisition matrix 128 x 128, in-plane-resolution 1.17 x 1.17 mm^2^, slice thickness 4 mm, number of excitations = 2, TA 2:53 min. Scans were repeated in case of motion artifacts.

### Electrical nerve conduction measurements

**Fig 1:**
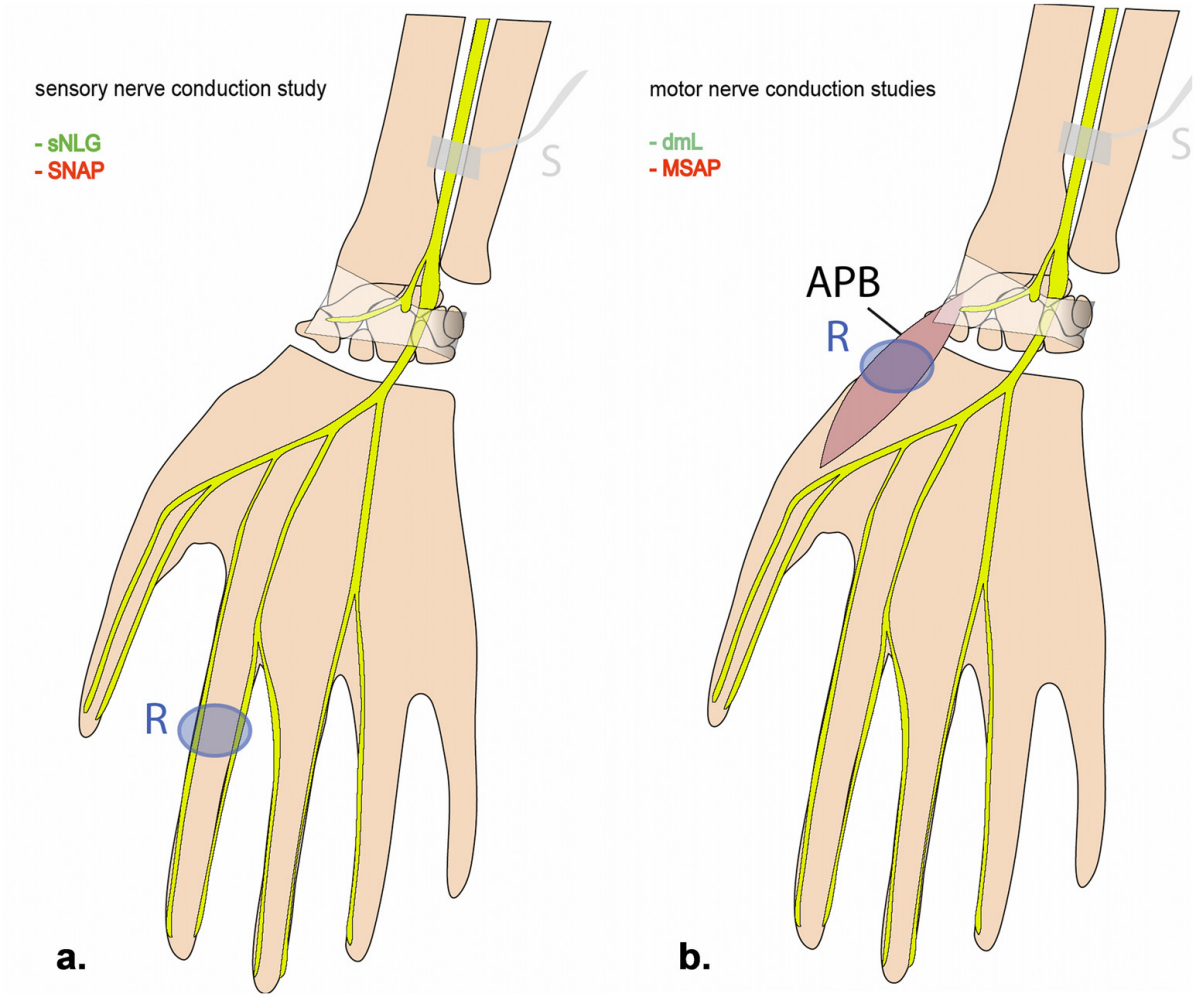
Schematic drawing of median nerve conduction measurements. Sensory nerve conduction study (a): stimulation is administered at the distal forearm; sensory nerve conduction velocity (sNCV) and sensory nerve action potential (SNAP) are recorded from the digital nerve of the index finger in antidromic technique. Motor nerve conduction study (b): stimulation is administered at the distal forearm; distal motor latency (dml) and compound muscle action potential (CMAP) are recorded from the APB. Electroneurographic parameters probing axon and myelin sheath integrity are printed in red and green, respectively. APB: abductor pollicis brevis muscle; S: stimulation site; R: recording site.

Prior to the MRI session, all subjects underwent electrodiagnostics for the median nerve at the wrist conducted by examiners with more than twenty years of experience. The obtained results were independently assessed by a board-certified neurologist and clinical neurophysiologist (MW) with more than eight years of experience in clinical neurophysiology. The wrist was placed in neutral position with the forearm in supination. Skin temperature was controlled for a minimum of 32°C. Acquired parameters included compound muscle action potential (CMAP) of the abductor pollicis brevis muscle as well as sensory nerve action potential (SNAP), distal motor latency (dml) and sensory nerve conduction velocity (sNCV) of the median nerve (see Fig 1). Reference values of the electrophysiological laboratory of the Department of Neurology at Heidelberg University Hospital were used as reference standard.

### Image analysis

DTI postprocessing and quantitative analysis was performed using FSL Diffusion Toolbox (FDT) of the FMRIB free software library (FSL, http://fsl.fmrib.ox.ac.uk/fsl) v5.0.6 (Oxford, UK) on an IBM PC with an Intel i7 processor running Ubuntu Linux v12.04.

The diffusion tensor model was fitted on the DWI images yielding maps of the first, second and third eigenvalues. From these images, parameter maps of fractional anisotropy (FA), mean diffusivity (MD), radial diffusivity (RD), and axial diffusivity (AD) were computed. Specifically, FA corresponds to a normalized standard deviation of the three eigenvalues (see standard formula in [32]), AD is identical to the first eigenvalue, RD equals the average of the second and third eigenvalue and MD equals the average of all three. MD is often referred to as apparent diffusion coefficient (ADC) in clinical routine.

Manual segmentations of the median nerve at the wrist were performed by two readers (AH, AX) on b = 0 images. Segmentations masks were created as overlays on b = 0 images using FSL's 'fslview' by labeling the median nerve in the central 12 slices of the acquisition volume thus covering the entire carpal tunnel and the distal forearm. Additionally acquired T2-w images were available for anatomical reference in cases of doubt. ROI placement was restrictive, which has been shown to result in observer-independent values [33]. Spatial registration of b = 0 images across subjects was performed by referencing to the hamulus of the hamate bone.

The segmentation masks of the median nerve were adopted to extract cross-sectional averages of DTI parameter values (FA, AD, MD, and RD) per slice and participant using customized FSL scripts. A moving average with a window width of three slices was applied on proximal-to-distal parameter profiles to increase signal-to-noise ratio.

### Statistical analysis

To assess interreader reliability, intraclass correlation coefficients (ICC) were computed per DTI parameter according to Shrout and Fleiss [34] and interpreted according to Landis and Koch [35]. If there is at least substantial interreader agreement as indicated by ICC values of 0.61 or greater, extracted parameter values from both segmentations are averaged in order to create a single dataset for further analysis. Otherwise, the individual segmentations are processed separately. ICCs were calculated using SPSS v15.0 (Chicago, IL, USA).

Pairwise Pearson correlations were computed between of median nerve DTI parameters (FA, AD, MD and RD, averaged across the carpal tunnel) and electroneurographic parameters (SNAP, CMAP, dml, and sNCV), yielding 16 correlations. The significance criterion was set at a p-value of 0.05 (one-tailed) applying Bonferroni correction for multiple comparisons. We imposed a one-tailed criterion due to our *a priori* expectation that electrophysiological measures would 'worsen' (i.e., decrease of conduction velocities and potential amplitudes) with reduction of tissue integrity as reflected by decreasing FA/AD and increasing MD/RD [22–29,36].

Data were controlled for outliers adopting Cook's Distance measure using the function 'regstats' in MATLAB v7.5 (Natick, MA, USA). Value pairs were suspicious of outliers when exceeding a Cook's Distance of 0.15, which computes according to the formula 4/(*n*-1-*k)*, where *n* is the number of participants (30) and *k* is the number of model parameters (2 for linear regression) [37]. Outliers were confirmed by visual inspection of scatter plots and removed from the data prior to correlation analysis.

Graphs mapping the proximal-to-distal course of nerve DTI parameters (FA, AD, MD, and RD) and scatter plots relating electrophysiology with DTI parameters were created using Microsoft Excel 2003 (Redmond, WA, USA). Statistical analysis was performed by AH and PB.

## Results

Interclass correlations for interreader assessment are shown in S1 Table. Agreement between both readers' median nerve segmentation was excellent. Readers' individual data per slice and DTI parameter are given in Table 1. Due to the high interreader agreement data from both readers were averaged for subsequent analyses. Electroneurographic parameters are provided in S2 Table. A high interindividual variation in electrophysiological measurements was observed and values were generally within the reference limits of the electrophysiological laboratory of the Department of Neurology at Heidelberg University.

**Table 1:** DTI parameter profiles of the median nerve per reader.

Reader	Variable	distance from hamulus (mm)									
		-28	-24	-20	-16	-12	-8	-4	0	4	8
Reader 1	FA	0.64 ± 0.12	0.62 ± 0.11	0.59 ± 0.10	0.56 ± 0.09	0.55 ± 0.08	0.54 ± 0.08	0.54 ± 0.07	0.56 ± 0.08	0.58 ± 0.07	0.59 ± 0.08
	AD (x10^-3^ mm^2^/s)	2.12 ± 0.16	2.13 ± 0.13	2.12 ± 0.13	2.12 ± 0.14	2.13 ± 0.19	2.16 ± 0.20	2.18 ± 0.19	2.20 ± 0.21	2.22 ± 0.24	2.23 ± 0.25
	MD (x10^-3^ mm^2^/s)	1.16 ± 0.23	1.18 ± 0.18	1.21 ± 0.15	1.24 ± 0.13	1.26 ± 0.13	1.28 ± 0.15	1.39 ± 0.15	1.28 ± 0.14	1.27 ± 0.15	1.27 ± 0.15
	RD (x10^-3^ mm^2^/s)	0.69 ± 0.29	0.71 ± 0.24	0.76 ± 0.19	0.79 ± 0.16	0.82 ± 0.16	0.84 ± 0.16	0.84 ± 0.16	0.83 ± 0.15	0.80 ± 0.15	0.78 ± 0.16
Reader 2	FA	0.66 ± 0.10	0.65 ± 0.10	0.61 ± 0.10	0.58 ± 0.10	0.55 ± 0.09	0.55 ± 0.09	0.55 ± 0.08	0.56 ± 0.08	0.58 ± 0.08	0.59 ± 0.08
	AD (x10^-3^ mm^2^/s)	2.09 ± 0.15	2.12 ± 0.14	2.12 ± 0.13	2.14 ± 0.14	2.14 ± 0.18	2.19 ± 0.20	2.19 ± 0.20	2.21 ± 0.20	2.21 ± 0.22	2.23 ± 0.23
	MD (x10^-3^ mm^2^/s)	1.11 ± 0.16	1.14 ± 0.15	1.18 ± 0.14	1.22 ± 0.13	1.26 ± 0.14	1.28 ± 0.14	1.29 ± 0.14	1.29 ± 0.14	1.27 ± 0.15	1.26 ± 0.15
	RD (x10^-3^ mm^2^/s)	0.63 ± 0.19	0.65 ± 0.20	0.71 ± 0.20	0.76 ± 0.19	0.82 ± 0.17	0.83 ± 0.17	0.84 ± 0.16	0.82 ± 0.16	0.80 ± 0.15	0.78 ± 0.16

Note.—Data are mean ± standard deviation. The position relative to the hamulus is indicated in millimeters (negative and positive values indicate position proximal and distal to that reference, respectively).

**Fig 2:**
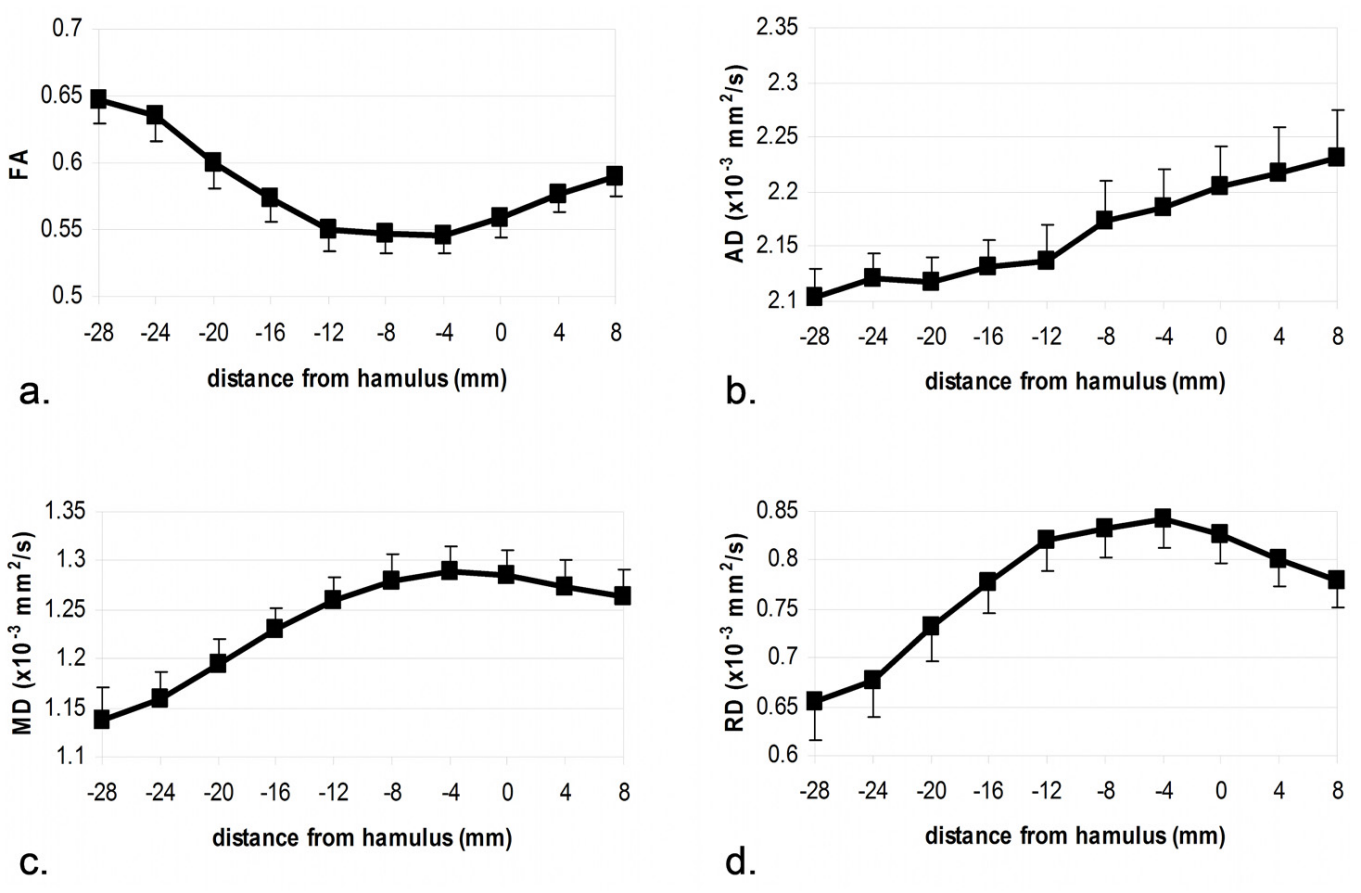
DTI parameter values of the median nerve at the wrist from proximal to distal. X-axis denotes the distance from the reference structure (hamulus of the hamate bone) in millimeters. Negative/positive values indicate position proximal/distal to that reference, respectively. Errorbars denote the standard error of the mean. FA: fractional anisotropy; AD: axial diffusivity; MD: mean diffusivity; RD: radial diffusivity.

Fig 2 displays proximal-to-distal DTI parameter values of the median nerve at the wrist (Fig 2). The FA profile is u-shaped with a minimum at -4 mm, i.e. four millimeters proximal to the hamulus of the hamate bone in the center of the carpal tunnel (Fig 2a). The RD profile appears as an inverted FA profile with the maximum again at -4 mm (Fig 2d). The MD and RD profiles appear similar with both maxima at -4 mm (Fig 2c and 2d). The extrema of FA, RD and MD are all distinct from the mean parameter baseline (t≥4.7(df = 29), p<0.001 for all parameters). Specifically, FA minimum was at -6.3% of mean baseline, and MD and RD maxima were at +8.0% and +15.3% of mean baseline, respectively. The AD profile differs markedly showing an almost linearly increasing proximal-to-distal gradient (Fig 2b). Example DTI parameter images of the median nerve within the carpal tunnel are shown in S1 Fig.

**Fig 3:**
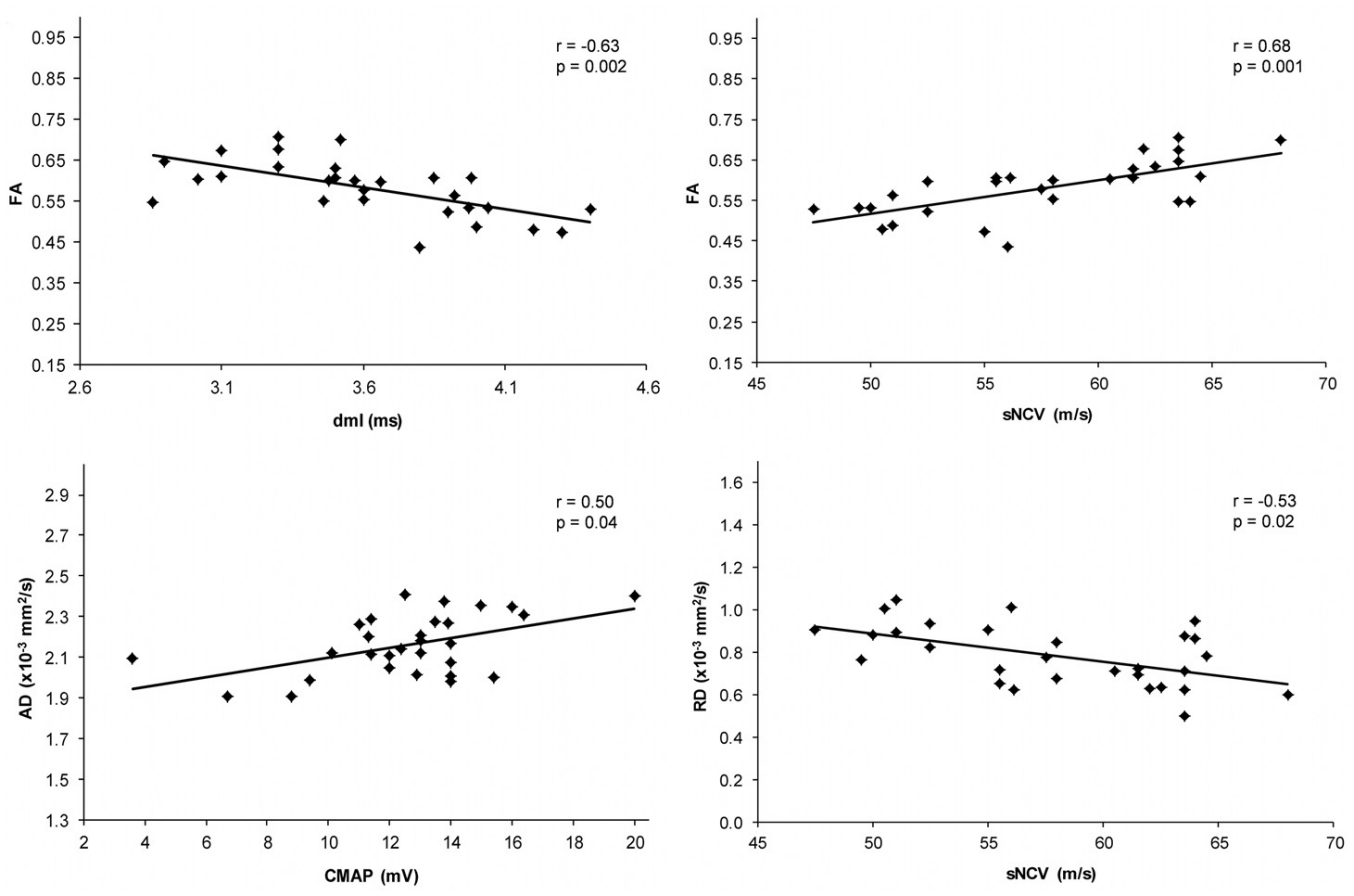
Scatter plots relating electroneurographic parameters with DTI parameters. Significant correlations surviving Bonferroni correction for multiple comparisons (see Table 2) are displayed. FA: fractional anisotropy; AD: axial diffusivity; RD: radial diffusivity; CMAP compound muscle action potential; dml: distal motor latency; sNCV: sensory nerve conduction velocity.

Correlation analysis between electroneurographic parameters and DTI parameters (Table 2) revealed a significant correlation between FA and electrophysiological measures of myelin sheath integrity. Specifically, FA correlated strongly with both distal motor latency (r = -0.63, p = 0.002, Bonferroni corr.) and sensory nerve conduction velocity (r = 0.68, p<0.001, Bonferroni corr.). There was also a moderate correlation with SNAP (r = 0.48), which was, however, not significant (p = 0.11, Bonferroni corr.). AD correlated significantly with CMAP as marker of axon integrity (r = 0.50, p = 0.04, Bonferroni corr.). AD did not show a meaningful correlation with SNAP (r = 0.14) and did not correlate with electrophysiological markers of myelin sheath integrity. MD did not show any significant correlation with electrophysiological parameters when applying strict statistical criteria. RD correlated with sensory nerve conduction velocity as marker for myelin sheath integrity (r = -0.53, p = 0.024, Bonferroni corr.). Correlation with the second marker of myelin sheath integrity, distal motor latency, was moderate (r = 0.46) but not significant applying strict statistical criteria (p = 0.10, Bonferroni corr.). RD did not correlate with electrophysiological markers of axon integrity. Scatter plots of the four significant findings are displayed in Fig 3.

**Table 2:** Correlations between electroneurographic parameters and DTI parameters.

Electrophysiological and DTI Parameters	FA		AD		MD		RD	
	r	p-value	r	p-value	r	p-value	r	p-value
SNAP	0.476	0.111 (0.007)	0.144	1.000 (0.232)	-0.125	1.000 (0.262)	-0.296	1.000 (0.067)
CMAP	0.107	1.000 (0.294)	**0.502**	**0.044 (0.003)**	0.205	1.000 (0.152)	0.115	1.000 (0.280)
dml	**-0.632**	**0.002 (<0.001)**	-0.221	1.000 (0.120)	0.212	1.000 (0.130)	0.459	0.099 (0.006)
sNCV	**0.680**	**0.001 (<0.001)**	0.084	1.000 (0.336)	-0.397	0.265 (0.017)	**-0.531**	**0.024 (0.002)**

Note.—Pearson's correlation coefficients and Bonferroni corrected p-values are shown. Uncorrected p-values are given in parentheses. Significant findings surviving Bonferroni correction for multiple comparisons are printed in bold. SNAP: sensory nerve action potential; CMAP compound muscle action potential; dml: distal motor latency; sNCV: sensory nerve conduction velocity.

## Discussion

In the present study we evaluated the potential of DTI parameters as imaging markers for axon and myelin sheath function in the median nerve. Even in healthy subjects, AD correlated significantly with an electroneurographic marker of axon integrity, while RD correlated with markers of myelin sheath integrity. These findings suggest that DTI may offer added value to conventional imaging techniques, which are unable to differentiate the axon compartment from the myelin compartment in peripheral nerves.

For the quantitative analysis, we continuously (slice-by-slice) mapped the proximal-to distal course of median nerve DTI parameters at the wrist. FA, MD, and RD profiles showed well-defined extrema suggestive of focal nerve alteration. Specifically, FA showed a minimum in the center of the carpal tunnel indicating a decrease in structural integrity. This finding is confirmed by RD and MD peaking at the same position suggesting reduction of myelin integrity and an increase in extracellular space, respectively. This coincidence of different DTI parameter extrema is indicative of a focal nerve alteration at a site where compressive neuropathy is frequently observed. As a typical entrapment site the carpal tunnel likely leads to inter-individually different degrees of median nerve affection without resulting in a manifest lesion, which is in accordance with electrophysiological studies [38–40].

At predilection sites for entrapment neuropathies, there is a broad spectrum of nerve alterations between healthy nerves at one end of the spectrum and clearly pathological nerves at the other end. Asymptomatic lesions with electrophysiological nerve conduction velocity slowing are frequently observed in clinical practice and have been reported in the literature in athletes or after physical compromise of a nerve [38–40]. Recently, subclinical lesions of the ulnar nerve at the ulnar groove at the elbow have also been shown by MRI with lowered FA values [21], analogous to the results shown here. However, in that study 10 of 30 participants showed pathological nerve conduction slowing, whereas in the present study electrophysiological parameters were generally within the range of normal reference values. Furthermore, we here aimed to differentiate nerval compartments, which was beyond the scope of the previous study. In this context our present findings suggest that DTI parameters including FA accurately capture the variance of electroneurographic parameters within physiological reference limits and therefore are truly "functional" imaging parameters, which are not limited to reflect clinically manifest structural lesions only.

DTI studies in humans rely on electroneurographic parameters for validation. This is straightforward since electrodiagnostics are routinely used to categorize nerve pathology as axonal or demyelinating. Specifically, decrease of nerve conduction velocity is primarily caused by segmental and paranodal demyelination and reactive remyelination as revealed by histopathological assessments [31,41]. Conversely, axonal degeneration leads to reduction of SNAP and CMAP amplitudes that are roughly proportional to the number of vital axons between recording and stimulation site [42]. Histopathologically, axonal degeneration is reflected by axonal swelling or disruption, cytoskeletal derangement and loss of fiber density [23,31,41,43,44]. With regards to diffusion tensor imaging there are few studies relating DTI metrics to electroneurographic parameters in peripheral neuropathies [18,20,45]. These previous studies aimed at a better understanding of the physiological underpinnings of DTI but did not yet address the question whether DTI is able to separately characterize the axon and the myelin compartment. Our study is the largest to date to systematically address that issue by investigating the correlation between full DTI read-out parameters and electroneurographic markers that probe both axon and myelin sheath integrity. Using electrodiagnostics for validation of candidate DTI metrics is warranted in this context, since peripheral nerve biopsies are rarely performed due to the inevitable functional loss, and electrodiagnostics are generally in good agreement with histopathological assessments of sural nerve biopsies in the evaluation of peripheral nerve injury as either axonal or demyelinating [31].

Applying strict statistical criteria, our results show that AD captures variance of an electrophysiological measure of axon integrity (CMAP), whereas RD captures variance of electrophysiological measures of myelin sheath integrity (dml, sNCV). Conversely, AD and RD show no reliable correlation with electrophysiological markers of myelin sheath and axon integrity, respectively. These findings confirm the role of AD and RD in the assessment of axons and myelin in CNS white matter tracts [22–27,29,36]. FA, an established integrity marker in peripheral nerves, correlates even stronger with dml and sNCV than does RD. This correlation of FA with electrophysiological markers of myelin sheath integrity is consistent with the notion that the myelin sheath is a significant modulator of FA in animal models [46] and in humans [47]. However, previous animal studies suggest that in case of manifest pathology, FA will likely also depend on axon integrity [46]. Of note, there is no meaningful correlation between AD and SNAP, which we assumed as the second marker of axon integrity (apart from CMAP). A reason might be that SNAP is more affected by temporal dispersion as caused by variance in nerve conduction velocities [42] than is CMAP. Thus, SNAP reduction is relatively ambivalent in that it may result from changes of both myelin sheath integrity (aggravating temporal dispersion) and axon integrity (impairing the number of action potentials). This may explain why FA exhibits a moderate (non-significant) correlation with SNAP, but not with CMAP that only correlates reliably with AD.

Our approach to study full DTI readout parameters allows investigating which parameters offer complementary information and which are possibly redundant. The parameter profiles indicate that the course of RD is very similar to FA (only inverted). MD reflects a mixture of AD and RD profiles, since MD mathematically is a combination of AD and RD. AD, however, differs markedly from FA and RD showing an almost linearly increasing gradient. AD also shows a completely different correlation structure with electrodiagnostics compared to FA/RD, whereas FA and RD are quite similar and MD is intermediate. These findings suggest that AD is a carrier of complementary information and should always be considered in future DTI studies. It has previously been shown in the CNS that loss of anisotropy may be caused by either increase in RD or decrease in AD (type 1 and type 2 anisotropy loss [9,22]) underscoring the importance of AD and RD for valid interpretation of FA.

The study group consisted of healthy participants only, which warrants the question about the sources of physiological variance in the data at hand. One source of variance is explained by the carpal tunnel being a strong predilection site for nerve entrapment. Due to pathophysiological considerations, mild nerve affection is likely to impact on the myelin sheath [48]. The severity and extent of this subclinical myelin damage likely varies across participants. Another source of physiological variance is age. Age dependency of both electrodiagnostics and diffusion metrics such as FA and ADC has been documented ([18,19]) and probably reflects degenerative changes in both the axonal and the myelin compartment as revealed by autopsy studies [49–52]. The age range in the study group was relatively wide (23–68 years) and age correction may be warranted when impact of age is considered as a confound (e.g., when patients and controls are not age-matched). In the healthy sample at hand, however, age may be regarded as a source of physiological variance facilitating meaningful correlations between the domains of electrodiagnostics and DTI. Still, so as to further elucidate the impact of age in our data, we removed the age effect using linear regression in a *post hoc* analysis. This is a very conservative approach, since age-dependent degenerative changes underlying variation in DTI parameter values are likely the same as those underlying variation in electrophysiological nerve function. Regressing out the age effect as a confound is therefore likely to remove variance of interest. The result is shown in S3 Table. Although correction for age effects led to a reduction in correlation strength as expected, the key correlation structure was nonetheless preserved suggesting that the DTI metrics AD and RD are valid biomarkers of axon and myelin sheath integrity independent of age.

AD and RD parameters are frequently assessed in the human brain but their interpretation is hampered by the presence of complex fiber architectures, such as crossing fibers [53,54], so that they are not used in clinical routine to differentiate between axon and myelin sheath injury. In contrast, interpretation of AD and RD in peripheral nerves with their relatively simple unidirectional structure appears straightforward and might allow routine assessment of axon and myelin integrity. Decreases in AD (axon disruption, cytoskeletal derangement) and increases in RD (degradation of myelin) reflect injury in these compartments as shown in animal studies of the CNS (e.g., [22,23]). Adopting RD and AD we discerned the two nerval compartments—the axon and the myelin sheath—in the median nerve at the wrist. Our results encourage further use of the technique in scientific and clinical investigations.

One limitation of this study is that we included only healthy participants. The diagnostic value of DTI parameters as biomarkers of axon and myelin integrity remains to be shown in clinically manifest neuropathies. For future studies the potential of DTI to discern axon and myelin lesions may be investigated in neuropathies that are either predominantly axonal (e.g., some chemotherapy associated neuropathies, such as those induced by vinca alkaloids or taxanes [55]) or predominantly demyelinating (e.g., multifocal motor neuropathy, MMN). Further, it is known that FA and ADC depend on age. Of note, key correlations between DTI metrics and electroneurographic parameters were retained even after removing the effect of age by linear regression. However, we chose not to include systematic age-correction, since we see age as a source of variance, not a confounder, in this particular study. Another limitation in the interpretation of this study is that we rely on correlation with electrodiagnostics for validation. Histology from sural nerve biopsies might be considered in future studies for validation, if available and representative for the neuropathy to be studied. Finally, in more severe neuropathies, changes in caliber and infiltration by cells may lead to unpredictable changes in DTI parameters. This in turn may affect the use of RD as imaging marker for myelin integrity and AD as marker for axonal integrity. Future studies with clearly defined neuropathies will need to address this point.

## Conclusion

In the present study we demonstrated the potential of DTI to differentiate between the axonal and the myelin sheath compartment in the median nerve. AD can serve as an imaging marker of axon integrity, while RD and FA can serve as imaging markers of myelin sheath integrity. DTI offers added value to conventional imaging techniques in MR Neurography, which are unable to differentiate both nerval compartments. Our results further support the notion that FA depends significantly on myelin sheath function. AD is an important carrier of information complementary to FA and RD. DTI parameters consistently indicated a focal decrease in integrity in the center of the carpal tunnel, likely representing a minimal lesion at a physiological entrapment site underscoring the high sensitivity of DTI. Future studies of clinically manifest peripheral neuropathies will be needed to evaluate the potential of DTI in providing biomarkers that differentiate axon and myelin sheath injury.

## Supporting Information

S1 Fig DTI parameter images of the median nerve at the wrist in an example subject. Cross-sectional slices of distal (a), central (b) and proximal (c) positions within the carpal tunnel are shown with magnification insets of the median nerve (panel B). Slice positions are indicated in the anatomical scheme to the left (panel A). FA and MD maps were calculated from the DTI sequence, colored FA maps (ColFA) encode the preferred diffusion direction (right<–>left = red, anterior<–>posterior = green, superior<–>inferior / perpendicular through plane = blue) and are generated by overlaying the principle diffusion vector of the diffusion tensor fit over the FA maps. MD: mean diffusivity map; FA: fractional anisotropy map; ColFA: colored FA map.
(TIF)

S1 Table ICC for interreader agreement per DTI parameter. (DOC)

S2 Table Results of electrodiagnostics per participant. (DOC)

S3 Table Age corrected correlations between electroneurographic parameters and DTI parameters. (DOC)
