# Supplementary figures and images for: Peripheral Nerve Diffusion Tensor Imaging: Assessment of Axon and Myelin Sheath Integrity
Source: PLoS One. 2015 Jun 26;10(6):e0130833. doi: 10.1371/journal.pone.0130833 (PMC4482724; doi:10.1371/journal.pone.0130833)

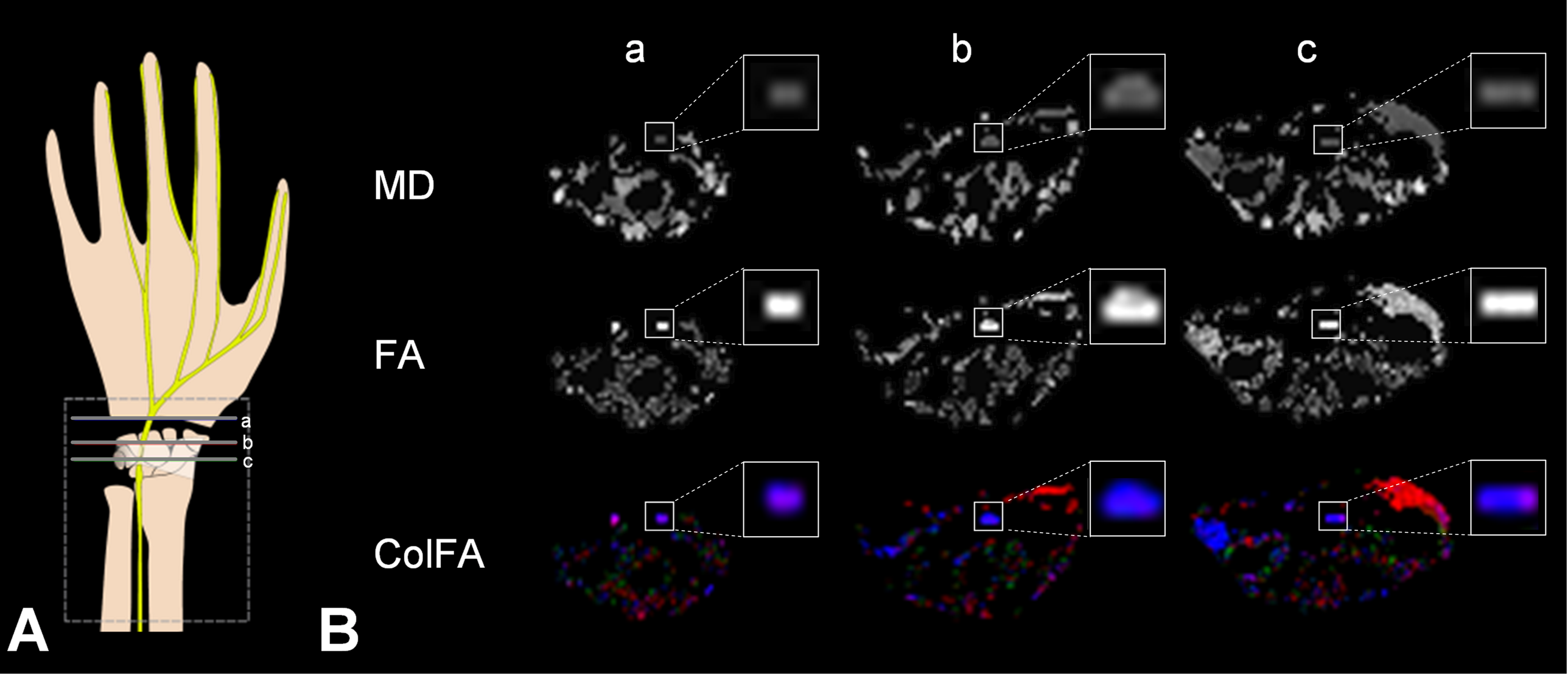

Supplement: S1 Fig — Cross-sectional slices of distal (a), central (b) and proximal (c) positions within the carpal tunnel are shown with magnification insets of the median nerve (panel B). Slice positions are indicated in the anatomical scheme to the left (panel A). FA and MD maps were calculated from the DTI sequence, colored FA maps (ColFA) encode the preferred diffusion direction (right<–>left = red, anterior<–>posterior = green, superior<–>inferior / perpendicular through plane = blue) and are generated by overlaying the principle diffusion vector of the diffusion tensor fit over the FA maps. MD: mean diffusivity map; FA: fractional anisotropy map; ColFA: colored FA map. (TIF) [file pone.0130833.s001.tif]
