# Supplementary material for: Peripheral Nerve Diffusion Tensor Imaging: Assessment of Axon and Myelin Sheath Integrity
Source: PLoS One. 2015 Jun 26;10(6):e0130833. doi: 10.1371/journal.pone.0130833 (PMC4482724; doi:10.1371/journal.pone.0130833)
Supplement: S1 Table — (DOC) [file pone.0130833.s002.doc]

| DTI Parameter | ICC |
| --- | --- |
| FA | 0.95 |
| AD | 0.94 |
| MD | 0.89 |
| RD | 0.90 |

Note.—ICCs were calculated according to Shrout and Fleiss and interpreted according to Landis and Koch . ICC of 0.01-0.20 indicates slight agreement; ICC of 0.21-0.40, fair agreement; ICC of 0.41-0.60, moderate agreement; ICC of 0.61-0.80, substantial agreement; and ICC of 0.81-1.0, almost perfect agreement.
