# Supplementary material for: Peripheral Nerve Diffusion Tensor Imaging: Assessment of Axon and Myelin Sheath Integrity
Source: PLoS One. 2015 Jun 26;10(6):e0130833. doi: 10.1371/journal.pone.0130833 (PMC4482724; doi:10.1371/journal.pone.0130833)
Supplement: S2 Table — (DOC) [file pone.0130833.s003.doc]

| Participant | SNAP (µV) | CMAP (mV) | dml (ms) | sensory NCV (m/s) | age (years) |
| --- | --- | --- | --- | --- | --- |
| 1 | 23.5 | 15.4 | 3.3 | 63.5 | 31 |
| 2 | 26.0 | 20.0 | 3.1 | 64.5 | 28 |
| 3 | 25.7 | 13.5 | 3.5 | 68.0 | 32 |
| 4 | 47.0 | 16.4 | 3.3 | 62.0 | 26 |
| 5 | 27.8 | 12.9 | 3.5 | 58.0 | 23 |
| 6 | 15.5 | 3.6 | 3.8 | 56.0 | 55 |
| 7 | 20.9 | 13.9 | 2.9 | 64.0 | 36 |
| 8 | 40.5 | 14.0 | 3.3 | 64.0 | 60 |
| 9 | 23.5 | 14.0 | 3.3 | 62.5 | 65 |
| 10 | 43.5 | 15.0 | 2.9 | 63.5 | 26 |
| 11 | 39.0 | 12.4 | 3.5 | 61.5 | 42 |
| 12 | 28.6 | 10.1 | 3.7 | 55.5 | 29 |
| 13 | 30.8 | 6.7 | 3.9 | 56.2 | 33 |
| 14 | 29.3 | 13.8 | 3.6 | 52.5 | 25 |
| 15 | 20.4 | 13.0 | 4.2 | 50.5 | 65 |
| 16 | 29.4 | 12.0 | 3.0 | 60.5 | 31 |
| 17 | 19.0 | 16.0 | 4.0 | 51.0 | 55 |
| 18 | 31.0 | 11.0 | 4.4 | 47.5 | 61 |
| 19 | 16.4 | 11.3 | 4.0 | 50.0 | 60 |
| 20 | 18.3 | 11.4 | 3.6 | 58.0 | 30 |
| 21 | 62.0 | 12.0 | 3.9 | 52.5 | 26 |
| 22 | 26.1 | 9.4 | 4.0 | 55.5 | 25 |
| 23 | 19.3 | 12.5 | 3.9 | 51.0 | 25 |
| 24 | 12.1 | 8.8 | 4.0 | 49.5 | 68 |
| 25 | 20.5 | 13.0 | 3.6 | 57.5 | 65 |
| 26 | 80.5 | 25.0 | 3.1 | 63.5 | 34 |
| 27 | 29.5 | 11.4 | 3.5 | 63.5 | 34 |
| 28 | 26.5 | 13.0 | 3.5 | 61.5 | 27 |
| 29 | 35.5 | 14.0 | 3.9 | 52.5 | 31 |
| 30 | 34.5 | 14.0 | 4.3 | 55.0 | 31 |
