# Supplementary material for: Peripheral Nerve Diffusion Tensor Imaging: Assessment of Axon and Myelin Sheath Integrity
Source: PLoS One. 2015 Jun 26;10(6):e0130833. doi: 10.1371/journal.pone.0130833 (PMC4482724; doi:10.1371/journal.pone.0130833)
Supplement: S3 Table — (DOC) [file pone.0130833.s004.doc]

| Electrophysiological and DTI Parameters | FA | | AD | | MD | | RD | |
| --- | --- | --- | --- | --- | --- | --- | --- | --- |
|  | r | p-value | r | p-value | r | p-value | r | p-value |
| SNAP | 0.310 | 1.000 (0.066) | 0.061 | 1.000 (0.381) | -0.041 | 1.000 (0.419) | -0.160 | 1.000 (0.217) |
| CMAP | 0.050 | 1.000 (0.402) | *0.480* | *0.077 (0.005)* | 0.258 | 1.000 (0.101) | 0.183 | 1.000 (0.180) |
| dml | **-0.546** | **0.026 (0.002)** | -0.168 | 1.000 (0.192) | 0.135 | 1.000 (0.243) | *0.365* | *0.447 (0.028)* |
| sNCV | **0.600** | **0.007 (<0.001)** | 0.074 | 1.000 (0.356) | *-0.345* | *0.574 (0.036)* | *-0.452* | *0.126 (0.008)* |

Note.—Age-corrected Pearson's correlation coefficients and Bonferroni corrected p-values (one-tailed) are shown. Uncorrected p-values are given in parentheses. Significant findings surviving Bonferroni correction are printed in bold. Significant findings uncorrected for multiple comparisons are printed in italics. SNAP: sensory nerve action potential; CMAP compound muscle action potential; dml: distal motor latency; sNCV: sensory nerve conduction velocity.
